# Extending the Population Health Workforce Through Service Learning Internships During COVID: A Community Case Study

**DOI:** 10.3389/fpubh.2021.697515

**Published:** 2021-06-21

**Authors:** Jeffrey Belkora, Tia Weinberg, Jasper Murphy, Sneha Karthikeyan, Henrietta Tran, Tasha Toliver, Freddie Lopez, Grant Tominaga, Michael Helle, Gina Intinarelli, Joshua Adler

**Affiliations:** ^1^Institute for Health Policy Studies, University of California, San Francisco, San Francisco, CA, United States; ^2^University of California, Berkeley, Berkeley, CA, United States; ^3^Office of Population Health and Accountable Care, University of California, San Francisco, San Francisco, CA, United States; ^4^UCSF Health, University of California, San Francisco, San Francisco, CA, United States

**Keywords:** service learning, population health, COVID-19, telehealth, undergraduate, navigation, internship, workforce innovation

## Abstract

This report arises from the intersection of service learning and population health at an academic medical center. At the University of California, San Francisco (UCSF), the Office of Population Health and Accountable Care (OPHAC) employs health care navigators to help patients access and benefit from high-value care. In early 2020, facing COVID-19, UCSF leaders asked OPHAC to help patients and employees navigate testing, treatment, tracing, and returning to work protocols. OPHAC established a COVID hotline to route callers to the appropriate resources, but needed to increase the capacity of the navigator workforce. To address this need, OPHAC turned to UCSF's service learning program for undergraduates, the Patient Support Corps (PSC). In this program, UC Berkeley undergraduates earn academic credit in exchange for serving as unpaid patient navigators. In July 2020, OPHAC provided administrative funding for the PSC to recruit and deploy students as COVID hotline navigators. In September 2020, the PSC deployed 20 students collectively representing 2.0 full-time equivalent navigators. After training and observation, and with supervision and escalation pathways, students were able to fill half-day shifts and perform near the level of staff navigators. Key facilitators relevant to success reflected both PSC and OPHAC strengths. The PSC onboards student interns as institutional affiliates, giving them access to key information technology systems, and trains them in privacy and other regulatory requirements so they can work directly with patients. OPHAC strengths included a learning health systems culture that fosters peer mentoring and collaboration. A key challenge was that, even after training, students required around 10 h of supervised practice before being able to take calls independently. As a result, students rolled on to the hotline in waves rather than all at once. Post-COVID, OPHAC is planning to use student navigators for outreach. Meanwhile, the PSC is collaborating with pipeline programs in hopes of offering this internship experience to more students from backgrounds that are under-represented in healthcare. Other campuses in the University of California system are interested in replicating this program. Adopters see the opportunity to increase capacity and diversity while developing the next generation of health and allied health professionals.

## Introduction: Description of the Nature of the Problem Being Addressed and Rationale for the Proposed Innovation

This case study reports on a collaboration that represents the intersection of two major trends: service learning in education (1–4) and population health in health care (5, 6). Service learning programs involve students in experiential learning outside of classroom settings. Population health programs target an entire population or panel of patients and attempt to address their health and wellness in an integrated and holistic fashion. The past decade has seen a steady increase in the proportion of patients cared for under accountable care or other risk sharing programs. Such programs create alignment for all parties for the provision of high quality and affordable health care, and create opportunity for health systems to innovate with new models of care delivery.

In early 2020, population health programs faced an influx of demand from patients who were potentially exposed to coronavirus infection and who needed help with testing, treatment, and tracing services related to COVID-19 (7). Meanwhile, undergraduate institutions have launched service learning programs to ensure that students are exposed to high impact practices such as internships (8–11). Students benefit from internships and other experiential learning opportunities because they allow students to apply knowledge, gain skills, interact with role models and mentors, and work on interprofessional teams (2, 12–14). Internships also present challenges, as well as opportunities, in terms of equity and access (15). This case study describes one such innovative internship at an academic medical center where students helped increase the capacity of a COVID hotline.

## Context (Setting and Population) in Which the Innovation Occurs

The setting for this case study was the University of California, San Francisco (UCSF). UCSF includes schools of medicine, nursing, pharmacy, and dentistry; as well as UCSF Health, a health delivery system. UCSF has over 26,000 employees and sees 45,000 hospital admissions and 1.7 million outpatient visits annually. Within UCSF, key parties to this case study included UCSF's Patient Support Corps (PSC) and the Office of Population Health and Accountable Care (OPHAC); and UCSF patients and employees concerned about obtaining testing, treatment, tracing, and return-to-work services relevant to COVID-19.

### The Office of Population Health and Accountable Care

In 2015, UCSF established its Office of Population Health and Accountable Care (OPHAC) to provide high-value care to defined populations of patients. In accountable care arrangements, public and private payers agree to share financial risk with a delivery system so that incentives are better aligned to focus on quality, not volume, of care. OPHAC employs 28 licensed professionals and 15 unlicensed navigators who provide care to 100,000 covered members. Along with analysts and other staff, this team helps patients access care related to cancer screenings, chronic obstructive pulmonary disease, depression, diabetes, end-stage renal disease, heart failure, hypertension, immunizations and other acute and chronic conditions.

In early 2020, in response to the pandemic, UCSF's Chief Medical Officer (author JA) tasked OPHAC with navigating patients and employees to established pathways for testing, tracing, treatment, and return to work. This led OPHAC to establish an employee- and patient-facing COVID telephone hotline. The volume of calls to this hotline quickly absorbed the capacity of existing staff navigators, who were redeployed from other population health assignments. Meanwhile, UCSF had implemented a hiring freeze. In hopes of expanding navigator capacity, OPHAC's director, author GI, responded to an email from UCSF's Chief Medical Officer (author JA), introducing OPHAC to author JB, director of the Patient Support Corps. In view of the COVID pandemic, JB had written to JA inquiring whether UCSF's pandemic response team could make use of student interns as workforce extenders.

### The Patient Support Corps

The Patient Support Corps is a service learning program in which UC Berkeley pre-health students serve as unpaid patient navigators while earning academic credit. A formal affiliation agreement between UCSF and UC Berkeley addressed parties' concerns about training, risk management, insurance, security, privacy and confidentiality, academic content, and academic credit. Since 2012, the Patient Support Corps has trained and deployed 137 undergraduates earning academic credit through UC Berkeley's Undergraduate Research Apprentice Program.

Until the 2020 pandemic, UCSF's participating clinical sites consisted of adult ambulatory care clinics. In 2020, the director (author JB) began a series of discussions with OPHAC leaders and staff about adding Population Health to the roster of clinical sites that host student interns.

### UCSF Patients and Employees Exposed to Coronavirus Infections (Hotline Callers)

The purpose of the Population Health collaboration with the Patient Support Corps was to better serve patients and employees exposed to Coronavirus infections. The institution directed patients and employees to call the COVID hotline to access testing, tracing, treatment, and return-to-work resources. OPHAC physicians authored branching logic instructions for hotline staffers to administer in guiding callers to the appropriate resources (16, 17). The instructions took the form of a base set of flowcharts, supplemented by a daily bulletin with modifications. Authors MH, HS, TT, GT, and FL made changes as new information emerged about the virus, testing, tracing, and treatment.

## Programmatic Elements

### Program Plan

In July 2020, OPHAC signed a memorandum of understanding with PSC, providing 1 year of financial support for the administration of the Patient Support Corps' recruitment, onboarding, training, deployment, and supervision of students. The PSC agreed to provide 15 students working half-day shifts on the COVID hotline. The agreement also foresaw that OPHAC would evaluate whether students could be deployed as workforce extenders for other population health tasks and assignments. Both sides entered the agreement hoping that the arrangement would lead to OPHAC becoming a long-term clinical site hosting PSC student interns.

Author JB articulated a program plan for the new internship. The program plan consisted of five sub-plans: the strategic direction (vision, purpose, mission, values, and goals); the service delivery plan (how callers would interact with student navigators); the operational plan (what resources students would access to deliver services); the evaluation plan; and the financial plan (18).

For the service delivery plan and operational plan, we relied on two principles of lean design: prototyping and iteration (19). The prototype phase consisted of having two students act as trailblazers during summer 2020, with the plan to expand the number in Fall 2020 based on lessons learned from the summer pilot. The iteration phase included recruitment and larger scale training for the Fall rollout; competency checking and deployment of students; supervised practice; and program expansion.

### Program Implementation

We now summarize, chronologically, our program implementation. In later sections, we will offer reflections on lessons learned.

#### Initial Training and Test Deployment

Author HT, a Clinical Manager in OPHAC, initially trained the two trailblazing students (authors JM and SK) on July 29, 2020. Topics included: the purpose of the hotline; the multiple workflows for patients calling regarding testing and symptom evaluation; the use of Cisco Finesse and Jabber telephony systems; and the documentation of telephone encounters on ApeX (UCSF's electronic health record) and Qualtrics (an online survey system). Author JB recorded this initial training so that future students could access it asynchronously.

##### Contents of Training on Service Delivery and Operational Plans

The initial training addressed the overall functioning of the COVID hotline as follows. The purpose of the UCSF COVID hotline was to answer caller questions about COVID-19, and to provide protocol-driven telephone triage and disposition for symptomatic and/or exposed UCSF patients. OPHAC created the hotline to refer callers to appropriate levels of care and direct them to resources such as the Center for Disease Control and San Francisco Department of Public Health website.

Callers called the UCSF COVID hotline and selected if they were a patient or employee. The Cisco Finesse system routed callers to a navigator who picked up the incoming call on their Cisco Jabber softphone interface.

For students to serve on the hotline, they needed to be onboarded, trained, and badged as UCSF affiliates. Students configured their personal laptops to comply with UCSF's information technology security requirements. They also installed enterprise software that encrypted their devices; provisioned them for remote wiping in case of theft or loss; and provided access to the Cisco Finesse and Jabber software applications, as well as UCSF's electronic health record and Qualtrics data collection platform.

Using Jabber, each student was able to transfer calls; merge two calls; place a call on hold; and message colleagues in the Jabber chat room. Navigators interacted with callers and followed scripts with branching logic created by physicians as part of a larger effort to triage patients and employees at UCSF (16, 17).

Navigators accessed the scripts on a shared Box folder. Hotline leaders updated a daily bulletin notifying navigators of changes, and changed the scripts or add new branching logic as needed, for example when a large employer contracted with UCSF for employee support. Hotline leaders hosted online meetings before the hotline opened each day to inform navigators of changes and gather feedback relevant to updating the scripts and branching logic.

Navigators documented each call in the patient's medical record by creating a telephone encounter record. In the documentation section of the telephone encounter, the navigator noted the patient's responses to the branching logic questions. Some outcomes of the branching logic resulted in the navigator routing the record of the telephone encounter to a nurse team or a scheduling team.

In addition to documenting their notes in the caller's electronic health record, navigators also entered data about each call into a data collection form hosted on the Qualtrics survey system. Here, navigators entered the caller's affiliation to UCSF; the main reason for calling the hotline; if the patient was adult or pediatric; if they were an employee; the patient's medical record number; the outcome of the call; and the name of the navigator completing the form.

##### Observation and Supervised Practice

On July 30 and 31, 2020, JM and SK devoted 6 h shifts to observing an experienced navigator take calls on the UCSF COVID patient hotline. On August 1 and 2, 2020, JM and SK simulated calls and protocols with each other, alternately playing the role of caller or hotliner. During the week of August 3, JM and SK fielded calls on the hotline under the observation of experienced health care navigators for 9 h while being given feedback on health care navigator competencies.

##### Formal Competency Check

The formal competency check consisted of experienced navigators, authors GT and FL, observing JM and SK take calls on the hotline via Zoom. GT and FL evaluated the students based on a competency checklist which included the measures listed below. If the intern successfully met these competencies, they were cleared to take calls independently:

- Box: locates Daily Bulletin, Schedule, and most recent Algorithms [scripts with branching logic], searches Daily Bulletin
- Jabber: Updates status w/phone number, sends message, creates group chat
- Language interpreting services: finds preferred language in chart, inserts SmartPhrase at top of note
- Transfer call to another agent
- Check eligibility: find a caller's Primary Care Physician or Specialist in their medical record
- Use of SmartPhrases in notes
- Use of Algorithm [script with branching logic] to determine disposition
- Routing notes about telephone encounter to Schedulers, with appropriate routing comments
- Qualtrics: properly documenting the call.

After passing the competency check, JM and SK began fielding calls independently on the hotline on August 11, 2020, completing over 100 h on the hotline through August 25, 2020.

#### Recruitment

Following the summer test, authors JB and TW proceeded with the plan to scale up in the Fall. Since 2013, the Patient Support Corps has recruited students in partnership with two UC Berkeley organizations. The first partner is UC Berkeley's Undergraduate Research Apprentice Program (URAP). Each semester, URAP allows researchers at UC Berkeley and other sites to post internship opportunities on a website portal accessible to UC Berkeley students. Students then apply through the portal, submitting essays and transcripts, and requesting to be interviewed. Those accepted then sign a learning contract and register for up to three units of academic credit in the course Undergraduate Interdisciplinary Studies 192. At the end of each semester, author JB assigns pass/fail grades based on student participation and performance in the program and in written and verbal critical reflections. Thus URAP is a crucial partner in the PSC's overall service learning endeavor, as it provides a mechanism for soliciting student applications, and registering students to earn academic credit via coursework.

The second partner in PSC recruiting is a student organization. In 2013, the first PSC students participating via URAP formed UC Berkeley's Patient Advocacy Student Group (PASG). Among other duties, student group leaders advertise the URAP application process; screen applicants; interview finalists; and recommend a slate of students for consideration by PSC leaders JB and TW.

The URAP deadline was August 31, 2020, at which point 269 applicants submitted their essays and transcripts for consideration. In consultation with JB and TW, JM and SK instructed the application reviewers to search for applications that demonstrated the following competencies.

Patient centered competencies:

- Humanism—caring about people as people, regardless of how different they may be;
- Remain neutral and focused on serving and advancing the patient agenda;
- Interact by telephone with clear volume and enunciation so that older or hard of hearing or Limited English Proficiency patients have the best chance of understanding;
- Paraphrase and summarize complex information concisely

Teamwork competencies:

- Follow complex instructions rigorously;
- Continuous improvement: discuss errors without covering up or worrying about losing face;
- Coordinate with other team members in pursuit of the mission, without worrying about who gets credit or who is noticed;
- Possibility of multi-year commitment as we put so much effort into training students

Pre-requisites included facility and flexibility with technology; and ability to type over 40 words per min.

Application reviewers used a Google Sheets document to record their notes and rankings of 269 applications. JM and SK identified 93 finalists to interview based on the application rankings. Interviewers completed interviews between September 4 and September 7, 2020, again recording their notes and ratings in a Google Sheet. On the evening of September 7, the interviewers convened online to discuss their impressions and ratings. After facilitating the process to consensus, JM and SK loaded the top-ranked applicants into 40 internship positions based on matching each candidate's availability with the internship shifts that needed to be filled. JM and SK assigned 11 of the 40 applicants into COVID hotline shifts. Nine returning student interns also slotted into COVID hotline shifts. Overall this process resulted in 10 pairs of students being slotted into morning or afternoon shifts for every day of the week, representing a capacity of 2.0 full-time equivalent navigators being added to the COVID hotline.

On September 8 and 9, PSC program leaders JB and TW called the applicants recommended by the student leaders to verify their suitability, offer them positions, and advise on next steps for accepting the positions. TW sent each new offeree, and all returning student interns from the previous year, the URAP learning contract and UCSF affiliate agreement for signature.

The learning contract specified the conditions for student participation, including the terms of the affiliation agreement between UCSF and UC Berkeley. By signing this contract, students agreed to devote 11 h a week to their internship responsibilities. The affiliate agreement included a Student Responsibility Statement, in which students agreed to follow all applicable regulations and policies, including those governing privacy and confidentiality (e.g., protecting patient information) and information technology security (e.g., using UCSF-encrypted devices for all program purposes.)

By September 11, all the offerees had accepted and signed their learning contracts and affiliate agreements. We then embarked on the task of training 18 students to join the two trail-blazing students on the COVID hotline.

#### Larger Scale Training

JM and SK led initial COVID hotline training sessions for 18 students on September 19th and September 26th 2020 via Zoom. Trainees first listened to the recording of the orientation given by HT about the purpose of the hotline, technology used, and workflows.

JM and SK then demonstrated how to field incoming calls on Cisco Finesse, use Cisco Jabber call functions, create and route a telephone encounter on the electronic health record system (ApeX), and track calls on Qualtrics.

Next, JM and SK reviewed the hotline caller workflows and demonstrated how navigators use the Cisco Jabber chat room to communicate in real-time with the nurse escalations team and consult with the hotline lead navigators.

On the second training day, JM and SK explained the various types of calls received on the hotline including non-UCSF patients inquiring about testing; patients calling to schedule their COVID test appointment; patients misdirected to the hotline; and patients exposed to COVID-19 positive individuals.

Students role-played the standard workflows and practiced documenting telephone encounters and using smart phrases with pre-populated questions in a test patient record. Finally, JM and SK assigned the interns to practice the workflows with a partner and submit a recording for evaluation.

Meanwhile, JM and SK expanded from their regular one shift to two half-day shifts per week on the hotline on 10/12, 10/19, and 11/2. They invited each new student to sign up for hourlong slots within these shifts. During these slots, JM and SK opened a Zoom session on their computer, sharing their sound and screen. In this way, the trainees could observe and hear JM and SK interacting with callers. Each new student observed JM and SK for a total of 1 h during the weeks of 10/5 and 10/12. Then JM and SK used Zoom to observe the trainees taking calls, for a total of 2 h each during the weeks of 10/19 and 11/2. JM and SK instructed trainees to place the patients on hold and consult with JM and SK when necessary. In complex cases, JM and SK could escalate questions to the Jabber chat room or message lead navigators.

#### Competency Checking and Deployment

As experienced navigators, authors FL and GT administered competency checks to determine whether trainees were ready to field calls on the hotline independently. FL and GT competency checked nine trainees the week of October 26, 2020, and approved six for independent work the week of 11/2. Since the 12 remaining trainees were not ready to field calls independently, they used their pre-assigned shifts time to practice alongside FL and GT or other experienced navigators on the hotline, either observing them or being observed via Zoom screen-sharing.

##### Supervised Practice During Suspension of Competency Checks

Between November 16 and December 10, 2020, lead navigators FL and GT suspended competency checks as they felt students needed more time observing and practicing. They suggested that students should shadow or be observed for 10 h before being competency checked. JM and SK arranged for students to practice under the supervision of the existing competency checked students, and to shadow the more experienced navigators on the hotline. Interns continued this training, spending ~5 h per week shadowing and precepting until the resumption of competency checks.

##### Resumption of Competency Checks

FL and GT resumed competency checking on December 11, 2020, and by December 31, 2020, 14 of the 20 interns were competency checked and able to take calls independently. By end of February, 2021, all 20 interns were taking calls independently. Through February, 2021, program records indicate that 20 interns worked 1,240 h on the hotline after being competency checked.

#### Program Expansion

##### Occupational Health

In mid-December, 2020, author MH identified the need to create a system for responding to employee questions about COVID exposure, testing, tracing, treatment, and return to work. Five students helped set up a process whereby employees could direct a voicemail or email message to UCSF's Occupational Health department, reporting contacts and exposures; positive test results; symptoms; adverse reactions to being vaccinated; or the need for return to work orders. Occupational Health navigators (including the 5 students) would then follow scripts and branching logic to either forward the emails or route notes about the voicemails to the appropriate Occupational Health staff who could address the employee questions. Five students devoted 270 h to Occupational Health between December 23, 2020 and January 21, 2021, a period corresponding to their winter break from school.

##### Population Health Outreach

In late 2020, the OPHAC shifted some navigator capacity from the COVID hotline back to regular care management activities, including outreach to patients with chronic conditions. Author JB contacted the Outreach Manager to see if students might help as workforce extenders in this arena. JB asked author SK to recruit one other student and work with the Outreach Manager to define a role for students in Population Health Outreach.

From January 7 to 11, 2021, the Outreach Manager and navigators oriented the two students to Population Health Outreach initiatives surrounding diabetes, child wellness, and hypertension. Then, from January 11 to 27, 2021, the students engaged in supervised practice with more experienced navigators. The students learned to conduct chart reviews to identify patients due for health maintenance exams, or who might be suffering from care gaps such as missing eye exams, blood pressure checks, and blood tests (e.g., Hemoglobin A1C lab). The students also learned how to schedule patients for appointments with a primary care doctor for diabetes follow up appointments. They learned how to pend orders for blood pressure cuff, HbA1C lab, and microalbumin lab and how to pend referrals to ophthalmology for diabetic eye exam.

Between January 27 and March 3, 2021, the students worked on outreach tasks relevant to patients with hypertension. They learn how to case-find and then contact patients for hypertension follow up, documenting their outreach encounter in the medical record, and scheduling patients for appointment in the next 2 weeks with their primary care physician. The students coached patients to bring their blood pressure medications, take blood pressure readings, and bring their blood pressure cuff to their appointment.

On March 3, 2021, author SK began independently performing chart review and outreaching to patients with diabetes, and the next week SK worked on screening and depression.

## Discussion Section That Shares Practical Implications, Lessons Learned for Future Applications

### Lessons Learned From Test Deployment

Our goal in launching with just two students was to test our program plan over the summer before scaling it up in the fall. This worked well. Key success factors included starting with two experienced students who could pair up for training, reflection, and peer support. However, using experienced students as trail-blazers meant that we underestimated the difficulty of training less experienced students. Also, hotline leaders had more bandwidth in the Summer to train two students than they did in the Fall to train 18 more.

### Lessons Learned From Recruitment

Our goal in the recruitment phase was to find 11 new students who would be immediately effective in joining 9 returning students, all learning to be COVID hotline navigators. This worked reasonably well but was extremely time consuming. To save time, we now recommend advertising 10 half-day shifts, and inviting candidates to apply for specific shifts. We could then save time by simply conducting interviews until we filled each shift. This search strategy is known as “satisficing”—spending resources (e.g., interviews) until a “good enough” solution is found, rather than interviewing all applicants to find the optimal candidates.

### Lessons Learned From Larger-Scale Training

Our goal in the larger-scale training was to get 18 students who were new to the COVID hotline up to speed without unduly burdening existing staff navigators. The hotline was experiencing very high call volumes and staff navigators were needed on the hotline. Therefore, we had to launch the navigators in waves rather than all at once. Now that the program is launched, each year we will have returning students who can serve as peer mentors and train new students, reducing the training burden placed on staff navigators. In addition, we plan in the future to institute a call recording feature. With other clinical sites, the Patient Support Corps has used call recordings to good effect as a way of accelerating training (20). For example, we have trainees transcribe calls, which is a way of having them absorb and reflect on dialogue in slow motion, through their fingertips.

### Lessons Learned From Competency-Checking and Deployment

Our goal with competency-checking and deployment was to ensure that only students who were well-qualified would be cleared to work independently on the COVID hotline. A key lesson here was that we attempted competency checking too early for some students who needed more time observing navigators, and practicing under supervision.

### Lessons Learned From Program Expansion

Our goal in the program expansion phase was to explore whether students could contribute to OPHAC in other internship roles, post-COVID. We conducted a test with the Outreach team, in which two students again blazed the trail by learning new and practicing new roles. From this test, we learned that students could contribute to outreach tasks, starting immediately with easier tasks (such as case-finding) and progressing to more challenging tasks (such as coaching patients to close key care gaps.) Overall, we concluded that the COVID hotline was an unusually intense way to launch a new program, as students had to be interacting in real time with patients in the stressful environment of a pandemic response. Deploying students to work on outreach tasks should be more straightforward. As author MH, Population Health Manager, said on a debriefing call, “We've proven that we can do this in a crisis. Now that we have time to actually develop something, we could do it 20 times better!”

### Implications for Future Service Learning Collaborations With Population Health Program

#### Next Steps at UCSF

We have identified some key next steps at UCSF. First, we are renewing and extending the memorandum of understanding for the collaboration between OPHAC and PSC. OPHAC will provide 3 years of funding to support part-time effort by the PSC faculty director and program coordinator. In turn, the Patient Support Corps will recruit, train, and supervise 20–40 students per year working to extend OPHAC's navigator capacity. We expect to bring on new students in their first or second year of college, and retain most students for 3 or 4 years.

Second, we are joining efforts to increase the diversity of students accessing our internship program. We will increase our outreach to pipeline programs, including those at local community colleges, to attract more students from under-represented backgrounds.

#### Opportunities for Other Population Health Programs

Each of the five medical centers in the University of California system has a Population Health program, and their leaders participate in a system-wide population health council. Student interns can provide an immediate boost to capacity, while also creating a pipeline of qualified candidates for future employment in these programs. Other academic medical centers outside of the UC system could likely benefit from similar internship programs, as could population health programs in community settings. The health care workforce must continue to evolve in order to reflect the diversity of our patient populations and to accommodate the novel ways in which patients will interact with the health care system. This program provides important training and a potential pipeline of future health care staff and professionals.

## Strengths, Limitations, and Conclusions

We have described the launch, under crisis conditions, of a novel population health internship program. This program increased the navigator capacity in our institution's population health program from 15 to 17 full-time equivalents—an increase of 13%. We relied on weekly group calls and *ad hoc* individual communications to monitor student growth. By these accounts, consistent with a prior study (21), students reported tremendous growth in core competencies including medical knowledge, patient care, systems-based practice, practice-based learning and improvement, interpersonal skills and communication, and professionalism. In future iterations of the program, we hope to record calls and survey students and callers as part of our evaluation.

In conclusion, our report provides a detailed description of a successful service learning internship program that contributed to our institution's COVID response. We are now investing in the continued expansion of this program. We anticipate that population health internships will contribute to increased capacity for delivering high-value care to patients; and that these internships can also help train more students from under-represented backgrounds in health care.

## Data Availability Statement

The raw data supporting the conclusions of this article will be made available by the authors, without undue reservation.

## Author Contributions

JB outlined and drafted the manuscript. TW, JM, and SK reflected on programmatic adaptations and other lessons learned from the Patient Support Corps perspective and contributed to corresponding sections of the manuscript. HT, TT, FL, GT, MH, and GI reflected on programmatic adaptations and other lessons learned from the Population Health perspective and contributed to corresponding sections of the manuscript. JA reflected on lessons learned from the institutional leadership perspective and contributed to the introduction and discussion. All authors contributed to the article and approved the submitted version.

## Conflict of Interest

The authors declare that the research was conducted in the absence of any commercial or financial relationships that could be construed as a potential conflict of interest.
